# A cost-effectiveness and value of information analysis to inform future research of tranexamic acid for older adults experiencing mild traumatic brain injury

**DOI:** 10.1186/s13063-022-06244-6

**Published:** 2022-05-03

**Authors:** Jack Williams, Katharine Ker, Ian Roberts, Haleema Shakur-Still, Alec Miners

**Affiliations:** 1 Department of Health Services Research and Policy, London School of Hygiene & Tropical Medicine, 15-17 Tavistock Place, London, WC1H 9SH UK; 2 Clinical Trials Unit, London School of Hygiene & Tropical Medicine, Keppel Street, London, WC1E 7HT UK

**Keywords:** Economic evaluation, Value of information, Head injury, Traumatic brain injury, Tranexamic acid

## Abstract

**Background:**

Tranexamic acid reduces head injury deaths in patients with CT scan evidence of intracranial bleeding after mild traumatic brain injury (TBI). However, the cost-effectiveness of tranexamic acid for people with mild TBI in the pre-hospital setting, prior to CT scanning, is uncertain. A large randomised controlled trial (CRASH-4) is planned to address this issue, but the economic justification for it has not been established. The aim of the analysis was to estimate the likelihood of tranexamic acid being cost-effective given current evidence, the treatment effects required for cost-effectiveness, and the expected value of performing further research.

**Methods:**

An early economic decision model compared usual care for mild TBI with and without tranexamic acid, for adults aged 70 and above. The evaluation was performed from a UK healthcare perspective over a lifetime time horizon, with costs reported in 2020 pounds (GBP) and outcomes reported as quality-adjusted life years (QALYs). All analyses used a £20,000 per QALY cost-effectiveness threshold.

**Results:**

In the base case analysis, tranexamic acid was associated with an incremental cost-effectiveness ratio of £4885 per QALY gained, but the likelihood of it being cost-effective was highly dependent on the all-cause mortality treatment effect. The value of perfect information was £22.4 million, and the value of perfect information for parameters that could be collected in a trial was £21.9 million. The all-cause mortality risk ratio for tranexamic acid and the functional outcomes following TBI had the most impact on cost-effectiveness.

**Conclusions:**

There is a high degree of uncertainty in the cost-effectiveness of tranexamic acid for older adults experiencing mild TBI, meaning there is a high value of performing future research in the UK. The value in a global context is likely to be far higher.

**Supplementary Information:**

The online version contains supplementary material available at 10.1186/s13063-022-06244-6.

## Background

Each year, about 69 million people sustain acute traumatic brain injuries (TBI) globally [1]. Around 90% are mild TBIs, as defined by a Glasgow Coma Scale (GCS) score of 13–15 [2]. Whilst those experiencing a moderate or severe TBI (GSC scores ≤ 12) are more likely to experience long-term disability or death following injury, there is still a risk of these outcomes following mild TBI [3, 4].

The CRASH-3 trial showed that tranexamic acid, given within 3 h of injury, reduces head injury deaths in people with moderate TBI and complicated mild TBI (defined as mild TBI with evidence of intracranial bleeding) [3]. Treatment was most effective when given soon after injury and only effective within 3 h. Indeed, in people with traumatic bleeding it is estimated that for every 15 min treatment is delayed, the survival benefit from tranexamic acid reduces by about 10% [5].

The CRASH-3 trial included people with mild TBI only if intracranial bleeding was present on a CT scan. The trial results raise the possibility that if patients were treated at the scene or on hospital arrival (without waiting for a scan), earlier treatment could prevent brain bleeding and result in improved health outcomes. Furthermore, the median time to CT scan for mild TBI is 3.7 h in England and Wales, meaning the majority of people with complicated mild TBI will be too late to benefit from tranexamic acid treatment even if bleeding is present [6].

The incidence of TBI is the highest amongst older adults, with the majority of these caused by falls [7, 8]. Older patients tend to have poorer outcomes following a TBI, with higher mortality rates, a higher probability of neurosurgery, and longer hospital stays [4, 9–11]. They are also more likely to experience intracranial bleeding, likely due to changes in anatomy, and a greater use of anticoagulant and antiplatelet drugs [12–14]. Due to the elevated risk of intracranial bleeding in older adults, there may be justification for tranexamic acid to be provided as a preventative treatment prior to onset of intracranial bleeding, rather than after it being observed, by which time it will usually be too late.

The CRASH-4 randomised controlled trial is planned to evaluate an intramuscular injection of tranexamic acid for symptomatic mild head injury in older adults, aged 70 and above (NCT04521881) [15]. The pilot phase of the trial has begun recruiting with a target of 500 patients, and funding is being sought for a full randomised controlled trial. This study is an early economic evaluation to evaluate the potential cost-effectiveness of tranexamic acid in older mild TBI patients, and to consider the potential treatment effects required for it to be cost-effective for mild TBI patients aged 70 and above.

There is currently no evidence around the effectiveness of tranexamic acid in this indication. However, the purpose of this economic evaluation is to identify the parameters that have the greatest influence on the cost-effectiveness, so that they can be prioritised for future research.

Furthermore, we have evaluated the value of further research, from an economic perspective. This approach assesses the uncertainty in a decision (i.e. whether or not to provide tranexamic acid to mild TBI patients), combined with the consequences of making a wrong decision, to estimate the monetary value that can be placed upon eliminating uncertainty [16–18]. The results are expressed in terms of the expected value of perfect information (EVPI), which represents the maximum amount that should be invested to eliminate all of the uncertainty in the decision model. If the cost of additional research, such as a new study, exceeds the EVPI, then the study does not represent value [17]. The EVPI can also consider the maximum value of research for individual or groups of model parameters, known as the expected value of partial perfect information (EVPPI) [17]. This analysis can help to identify the maximum value that should be spent on studies of specific parameters. This can help to identify parameters where there is a high value to eliminate the uncertainty within them, which should therefore be prioritised in any future studies, whilst also identifying where additional research for specific parameters is of less value.

## Methods

### Analysis

An economic model was developed to assess the cost-effectiveness of providing tranexamic acid to people following mild TBI. The model compared the current standard of care with and without the addition of tranexamic acid, for the treatment of older adults with mild TBI. For the model, the mean age of mild TBI patients was assumed to be 80 years old, since the pilot CRASH-4 trial is recruiting people aged 70 and above. We also report the cost-effectiveness for patients with a mean age of 60, 70 and 90 years old, since the incremental costs and benefits associated with treatment vary with age.

The analysis was performed from a UK NHS health service perspective. The model captures costs and outcomes over a lifetime time horizon. Outcomes are presented as quality-adjusted life years (QALYs) and costs are presented in 2020 pounds (GBP), both discounted at 3.5%, as recommended by The National Institute for Health and Care Excellence (NICE) [19]. The incremental cost-effectiveness ratio (ICER) per QALY gained was estimated by dividing the incremental costs associated with tranexamic acid by the incremental outcomes.

### Model structure

The economic analysis was performed using a simple Markov model with two health states; alive and dead. The model structure is similar to that used in a previous cost-effectiveness analysis of tranexamic acid in TBI patients (Additional file 1: Fig. S1) [20]. The model has a cycle length of 1 month for the first year, to capture the high risk of death in the first month post-injury. Thereafter, an annual cycle length is used for the remainder of the lifetime time horizon. The model was stopped once the cohort reached 100 years of age.

### Model parameters

#### Risk of head injury death

Numerous studies report the risk of death amongst older adults sustaining mild TBI. A meta-analyses in older adults reported in-hospital mortality risk ranging from 2.9 to 14%, but these tended to be older studies and some selected a more severe set of mild TBI patients (e.g. those with a hospital stay > 24 h) [11]. Three other studies were identified that reported in-hospital mortality for older adults ranging from 1.6% (amongst over 50s), to 3.7% (amongst over 65s) [21–23]. In the base case analysis, we used a weighted average of these three studies, with a risk of 2.1%. We used a triangular distribution from 1 to 5% to account for the high degree of uncertainty around this parameter.

#### Risk ratio treatment effect for in-hospital mortality (within 28 days)

In the absence of a randomised trial in this specific population, we used the treatment effect for mild TBI patients with intracranial bleeding from the CRASH-3 trial for the base case analysis [3]. The risk ratio for all-cause mortality within 28 days of injury was 0.70 (95% CI 0.45–1.08). As such, the risk ratio was applied for the first month of the model, after which the mortality risk was assumed equal for both treatments. Given that the treatment effect was derived from mild patients with intracranial bleeding, there may be a smaller treatment effect in all mild patients. We therefore evaluated the impact of mortality risk ratio treatment effects of 0.8 (0.52–1.24), 0.9 (0.58–1.39) and 0.95 (0.62–1.47). These scenarios assumed the same variance around the central estimate.

#### Post-discharge risk of death

Following the initial risk of death in the first month following mild TBI (i.e. in the first model cycle), a lifelong increase in the risk of death post injury compared to the general population was applied based on a case-control study from Scotland, with the risk derived from those older than 54 years [24]. A standardised mortality ratio (SMR) of 2.89 (2.36–3.54) was used in the first year post-injury, and 1.59 (1.27–1.91) thereafter, for both treatments. Although this study included people with moderate and severe TBI, there was no association between TBI severity and the increased risk of death, with the analysis stating that death rates were still twice as high following mild TBI.

#### Adverse events

Despite the CRASH-3 trial finding no evidence of any increase in adverse events associated with tranexamic acid, these were included in the model for both treatment groups, to account for the uncertainty around such events in this indication. The baseline probabilities for adverse events were derived from complicated mild TBI patients in the CRASH-3 study, since some probabilities differed by TBI severity. The risk ratio of events for tranexamic acid versus placebo was derived from all CRASH-3 patients, as we assumed the relative differences would be similar [3]. We estimated a cost associated with each adverse event, but did not include any utility decrement. To avoid double counting, we did not assume any additional length of hospital stay or mortality risk for adverse events.

#### Utility

Utility values for mild TBI were derived from a systematic review and mapping study [25]. This study reported utility values specific to the clinical outcomes following TBI, based on the Glasgow Outcome Scale (GOS). We combined these utility values with GOS outcomes reported 6 months post-injury in a study of patients hospitalised with mild TBI (of all ages) [26]. The mean utility for all mild TBI patients was estimated at 0.796 for those surviving post-TBI, which was equal for both treatment groups. Differences in these utility values between treatments were considered in deterministic threshold analyses. An age-based, general population utility decrement was applied to this mild TBI utility value, to capture the reduction in utility with age [27].

#### Costs

The cost of giving tranexamic acid included the cost of the drug, the needle and the syringe (Table 1). We assumed treatment administration would cost £4, based on an assumed 5 min to draw and administer treatment by a band 6 paramedic, with an hourly cost of £48 [28]. The mean cost of 500 mg tranexamic acid solution for injection was £1.50, derived from the British National Formulary [29]. The cost of a needle and syringe was £0.12 [30].

**Table 1:** Base case parameter values and distributions

Parameter	Value	Distribution	Source
**Clinical**			
Risk of death following head injury (first month post-injury)	0.0208	Triangular (0.01–0.05)	[21–23]
Tranexamic acid treatment effect on death following head injury	0.70	Lognormal (95% CI: 0.45–1.08)	CRASH-3 trial data
Standardised mortality ratio (first year*)	2.89	Normal (95% CI: 2.36–3.54)	[24]
Standardised mortality ratio (after first year^)	1.56	Normal (95% CI: 1.27–1.91)	[24]
**GOS outcomes**			
Good recovery	208	Dirichlet (208, 49, 23, 5)	[26]
Moderate disability	49	Dirichlet (208, 49, 23, 5)	[26]
Severe disability	23	Dirichlet (208, 49, 23, 5)	[26]
Vegetative state	5	Dirichlet (208, 49, 23, 5)	[26]
**Utility**			
Good recovery	0.894	Beta (*α* = 4331, *β* = 513.5)	[25]
Moderate disability	0.675	Beta (*α* = 2481.5, *β* = 1194.8)	[25]
Severe disability	0.382	Beta (*α* = 662.2, *β* = 1071.3)	[25]
Vegetative state	−0.178	Normal (95% CI: −0.330, −0.026)	[25]
Average utility (weighted average of utility and GOS outcomes)	0.796	By component (above)	
**Treatment-related costs**			
Tranexamic acid	£1.50	N/A	[29]
Needle and syringe	£0.12	N/A	[30]
Treatment administration time (minutes)	5	Uniform (2–8)	Assumption
Paramedic cost (per hour)	£48	N/A	[28]
**Hospital-related costs**			
Cost of neurosurgery	£7780	N/A	[32]
Proportion receiving neurosurgery	3.45%	Beta (*α* = 23.83, *β* = 667.01)	[4]
Initial hospital cost	£476	N/A	[32]
Additional hospital cost (per day)	£328	N/A	[32]
Mean length of stay	4 days	Gamma (*k* = 32, *θ* = 0.125)	[31]
**Adverse event costs**			
No tranexamic acid	£27.62	See Additional file 1 for distributions by component.	[3, 32]
Tranexamic acid	£28.72	See Additional file 1 for distributions by component.	[3, 32]
**Monitoring costs—first year**			
Good recovery	£303	Gamma (*k* = 25, *θ* = 9.6) × Inflation index*	[37, 38]
Moderate disability	£21,633	Gamma (*k* = 25, *θ* = 686.4) × Inflation index*	[37, 38]
Severe disability	£42,737	Gamma (*k* = 25, *θ* = 1356) × Inflation index*	[37, 38]
Weighted average (weighted by GOS score)	£8,139	Calculated by component	
**Monitoring costs—after first year**			
Good recovery	£27	Gamma (*k* = 25, *θ* = 0.96) × Inflation index^*^	[38]
Moderate disability	£1784	Gamma (*k* = 25, *θ* = 64) × Inflation index^*^	[38]
Severe disability	£13,934	Gamma (*k* = 25, *θ* = 500) × Inflation index^*^	[38]
Weighted average (weighted by GOS score)	£1695	Calculated by component	

*Costs are presented in 2019/2020 values. Inflator for first year post discharge costs (2006/2007 to 2019/2020) is 1.261, for post-discharge costs beyond the first year (2011/2012 to 2019/2020) is 1.115, and for costs sourced from NHS reference costs (2017/18 to 2019/20) is 1.046

The mean length of stay for people with mild TBI was assumed to be 4 days, based on a study in Australia of patients over 65 experiencing mild TBI [31]. The daily cost of hospitalisation was derived from NHS reference costs, to give an overall hospital cost of £1,711 [32]. A systematic review and meta-analysis estimated 3.5% of patients experiencing complicated mild TBI undergo neurosurgery [4]. The cost of neurosurgery was estimated to be £7440, derived from NHS reference costs, and this cost was additional to the hospital length of stay cost [32].

After discharge, survivors were expected to accrue post-discharge hospital costs, relating to their TBI. These costs were weighted by GOS score and different in the first-year post-discharge, and beyond. The mean annual costs were £7805 in the first year and £1626 thereafter.

The costs of adverse events were derived from NHS reference costs [32]. Day case reference costs were used to avoid double counting any additional hospital length of stay. The mean cost of adverse events for each individual receiving placebo and tranexamic acid was £27.62 and £28.72 respectively (Table 1). The breakdown of adverse events and costs are shown in Additional file 1. All costs in the model were inflated to 2020 costs, using the NHS inflation index [28].

### Model analyses

A willingness to pay threshold of £20,000 per QALY was adopted, as this is the lower bound of the NICE Technology Appraisals cost-effectiveness threshold, applicable in England and Wales [19].

Deterministic threshold analyses considered the potential treatment effects of tranexamic acid that would be required for treatment to be cost-effective (at £20,000 per QALY). The model explored the following potential treatment effects: relative risk of death (first month post-trauma), utility increment (assumed to apply for 1 month only), absolute reduction in hospital length of stay or relative reduction in neurosurgical intervention.

A probabilistic sensitivity analysis was performed by running 10,000 model simulations, with values for each probabilistic parameter (reported in Table 1) drawn from the relevant distribution. An analysis of covariance (ANCOVA) was performed using the results of the probabilistic sensitivity analysis, to understand the contribution of each probabilistic parameter on the overall uncertainty estimated in the model.

The EVPI represents the maximum value to eliminate uncertainty in the cost-effectiveness decision and therefore avoid the possibility of making the wrong decision. This was estimated using the results of the probabilistic sensitivity analysis simulations. The EVPPI was also estimated for those parameters that would be expected to be collected in a randomised trial, such as the planned CRASH-4 trial. This included the risk of death following mild TBI, the tranexamic acid treatment effect, patient outcomes at 1 month (or discharge), hospital length of stay, the proportion of patients receiving neurosurgery, the treatment administration time and the probabilities for adverse events. It did not include post-discharge costs, the utility values for each GOS outcome, or the SMR values applied to the long-term risk of death following mild TBI, since these would be unlikely to be collected in the trial. EVPPI loops were also performed for the above groups of parameters, to show the value of perfect information for these parameter groups. All EVPPI analyses were performed using a double-loop Monte Carlo method [33]. The EVPPI analysis of trial parameters were performed using 10,000 outer loop and 1000 inner loop simulations. For the EVPPI analysis of individual parameters, we performed 1000 inner loop and 1000 outer loop simulations, due to the high computation time to run the simulations. A higher number of outer loops were performed for the EVPPI analysis of trial parameters since the majority of the uncertainty was within these outer loops (e.g. the trial parameters), and an aim was to specifically compare the EVPPI for trial parameters with the EVPI analysis which used 10,000 simulations.

These analyses provide the value of information per patient, and this result was multiplied by the estimated UK population that could benefit from treatment. An English study of hospital admissions in 2002/2003 reported an incidence of TBI of 410.8 per 100,000 for those aged 75 and above, and it was assumed that 90% of these were mild TBI [2, 8]. Since the incidence in older groups has increased over recent years, we used this for all patients 70 and above [34]. This was multiplied by the UK population aged 70 and above, projected forward (from 2022 to 2041) over a 20-year period in which patients were assumed to benefit from treatment [35]. EVPI results are also presented for 5-, 10- and 30-year time horizons. Future benefits were discounted at 3.5%. In an analysis which also considered the value of information for those age 60 and above, we assumed the incidence was 50% lower from 60 to 69 years of age than for those 70 and above.

The economic model was developed in R v4.0.5 and is available online. A replicate deterministic model was also developed in Microsoft Excel for validation.

## Results

### Base case model results

In the base case analysis, providing tranexamic acid resulted in incremental costs of £96.69 and incremental QALYs of 0.0198 versus no tranexamic acid (Table 2). This resulted in an ICER of £4885 per QALY gained.

**Table 2:** Base case cost-effectiveness results

Treatment	Costs	QALYs	Incremental Costs	Incremental QALYs	ICER (QALY)
No tranexamic acid	£16,019	3.0656			
Tranexamic acid	£16,115	3.0854	£96.69	0.0198	£4,885

When considering the impact of age, the ICER was lower at younger ages, when assuming a constant risk of mortality following mild TBI (see Additional file 1: Table S2).

### Deterministic threshold analyses

We explored the potential treatment effects required for tranexamic acid to be cost-effective. Tranexamic acid would remain cost-effective with a mortality risk ratio of 0.993, a utility of 0.004 higher for tranexamic acid for 1 month compared to no tranexamic acid, a mean reduction of 0.02 days in the hospital length of stay, or a relative risk of neurosurgery of 0.974 (a 2.6% relative reduction). We also found the mortality treatment effect required for tranexamic acid to be cost-effective was slightly more modest at younger ages (Additional file 1: Table S3).

### Probabilistic sensitivity analysis

**Fig. 1:**
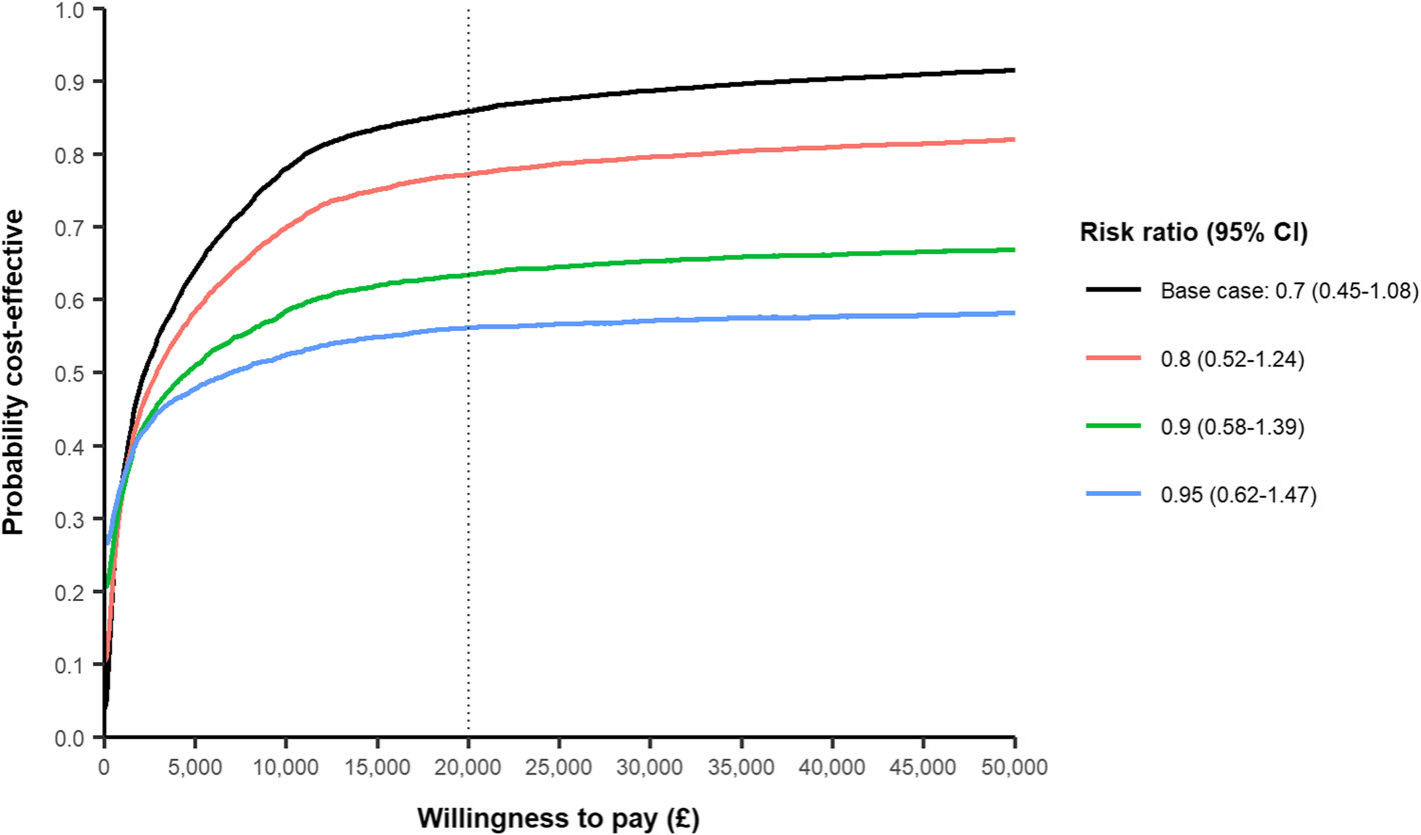
Cost-effectiveness acceptability curve (CEAC) based on the results of the base case probabilistic sensitivity analysis

The probabilistic sensitivity analysis showed that treatment was 86% likely to be cost-effective at £20,000 per QALY gained in the base case probabilistic analysis, with a mean ICER of £4,946 per QALY. Tranexamic acid was less effective than no tranexamic acid in 5.6% of simulations (Fig. 1). When considering more modest mortality treatment effects (risk ratio from 0.8 to 0.95), the probability of cost-effectiveness lowered, ranging from 56 to 77% (Fig. 1).

**Fig. 2:**
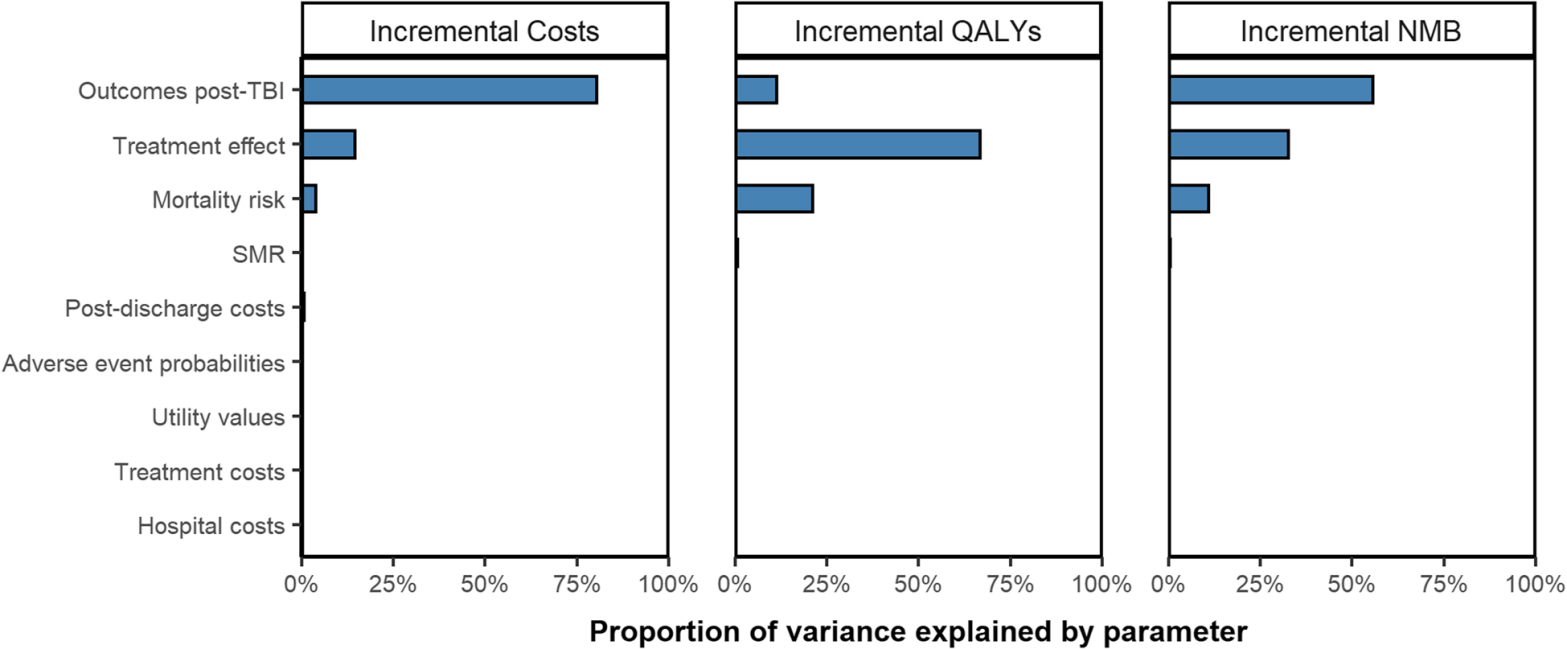
Analysis of covariance (ANCOVA) to show the variability in the model outcomes explained by the uncertainty in the groups of probabilistic parameters in the model. The model outcomes shown are the incremental costs, incremental QALYs and incremental net monetary benefit (NMB) at a £20,000 per QALY threshold

The ANCOVA analysis showed that most of the variability in the incremental costs, incremental QALYs and incremental NMB was due to the uncertainty in two parameters: the outcomes following mild TBI and the tranexamic acid mortality risk ratio (Fig. 2). The risk of mortality following head injury also had a small effect on the incremental outcomes.

### Value of information analyses

**Fig. 3:**
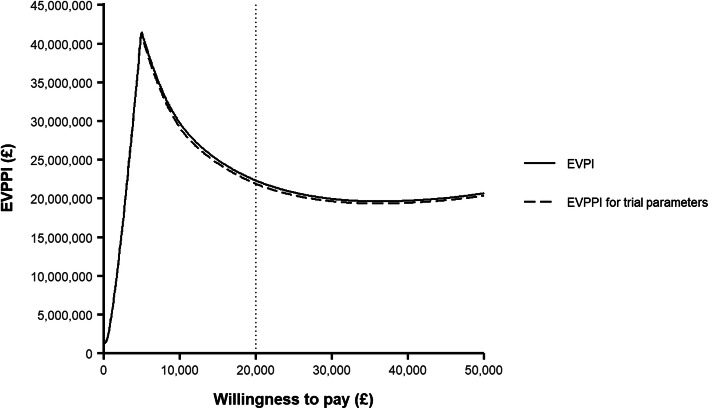
Expected value of perfect information (EVPI) for all parameters and expected value of partial perfect information (EVPPI) for parameters that could be collected as part of a prospective trial design

In the base case analysis, the EVPI was £37.06 per individual at the £20,000 per QALY willingness to pay threshold. This gave an estimated £22.4 million for the eligible population, over a 20-year time horizon (Fig. 3).

**Fig. 4:**
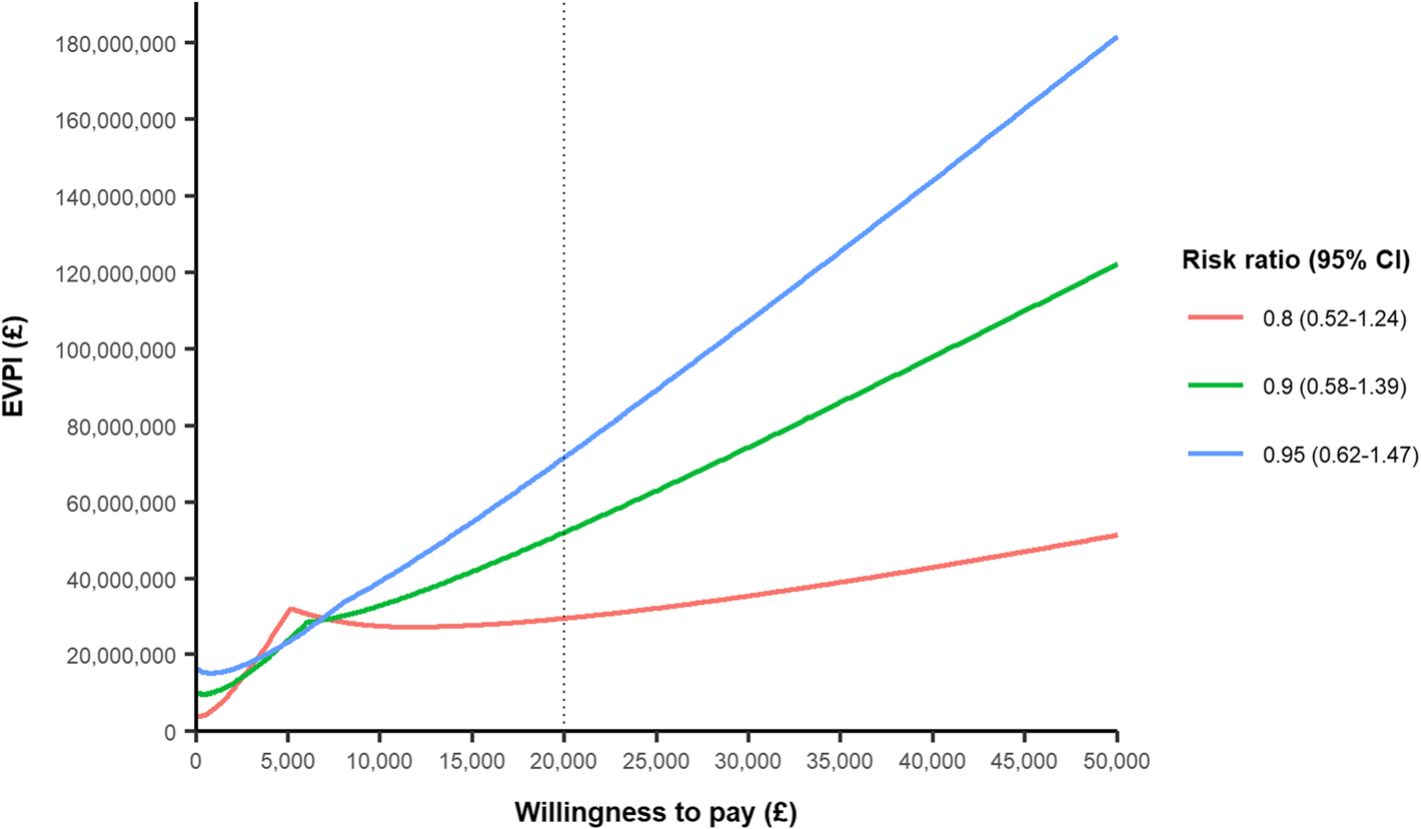
Expected value of perfection information across a range of risk ratio treatment effects. The sensitivity analysis assumes the same variance around the risk ratio as used in the base case analysis

The EVPI also increased when considering more modest mortality risk ratio treatment effects. At a mortality risk ratio of 0.95 (0.62–1.47), the EVPI increased to £71.7 million (Fig. 4). This is due to greater uncertainty in the decision at more modest treatment effects. In a scenario considering an eligible population aged 60 and above, with a mean age of 70, the EVPI increased to £43.6 million.

We also evaluated the EVPPI for all parameters planned to be collected in the CRASH-4 trial (including the risk of death following head injury, relative risk of death with tranexamic acid, outcomes of those with mild TBI, the proportion receiving neurosurgery, the mean length of stay and the probability of adverse events). The EVPPI for all trial parameters was £21.9 million at the £20,000 threshold (Fig. 3).

The EVPPI for each individual set of parameters was the highest for the post-TBI outcomes, and for the mortality risk ratio tranexamic acid treatment effect (see Additional file 1: Fig. S3). The value for other parameters was zero at the £20,000 threshold.

## Discussion

### Main findings and interpretation

There have been no reported trials or economic evaluations of tranexamic acid for all patients sustaining a mild TBI. The aim of this analysis was to consider whether tranexamic acid could be cost-effective for older mild TBI patients without the need for a prior CT scan and consequent delay in receiving treatment. Our base case analysis suggests that whilst this is more likely than not, there is sufficient uncertainty to warrant further clinical research. Indeed, the two parameters that had the most influence upon the results were the patient outcomes following mild TBI (which informed the utility values and post-discharge costs), and the mortality risk ratio associated with tranexamic acid, both of which should be prioritised in any future trial, such as the CRASH-4 trial.

In our base case analysis tranexamic acid could be cost-effective even with a very modest treatment effect, since the treatment is inexpensive and easy to administer. Even if tranexamic acid does not reduce mortality in mild TBI patients, it may be cost-effective if it leads to marginally higher utility amongst surviving patients, a small reduction in hospital length of stay or a lower proportion of patients requiring neurosurgery (Additional file 1: Table S3).

Since there remains uncertainty around the cost-effectiveness of treatment for mild TBI patients, there is a high value for additional research, to avoid the potential costs (financial or negative health outcomes) of making the wrong decision about whether treatment for this indication should be routinely recommended. In the base case analysis, the value of perfect information for parameters that could be collected within a randomised controlled trial is £21.9 million, which is likely to be far higher than the expected cost of the CRASH-4 trial. Given the lack of empirical evidence for the treatment effect in this indication, the value of information results were even higher when considering more modest treatment effects. Our results suggest the trial should focus on the mortality risk ratio treatment effect for tranexamic acid, as well as the patient outcomes (i.e. GOS outcomes) following mild TBI, which can be used to estimate quality of life and healthcare costs post-discharge. Other potential treatment effects not included in our base case analysis, such as differences in the disability or quality of life between groups following mild TBI, or differences in hospital costs, would also likely have a large impact on cost-effectiveness.

Tranexamic acid was marginally more cost-effective amongst younger patients, since the life expectancy for each life saved was longer, resulting in more QALYs. If the trial inclusion criteria were to be expanded to include those aged 60 and above, then the EVPI would increase to approximately £43.6 million, primarily due to the larger eligible population that would benefit from the treatment decision.

The EVPI values should be considered uncertain themselves, given the lack of empirical data upon which the treatment effect was based. Furthermore, these EVPI values should also be considered conservative for several reasons. Firstly, it assumes that only those aged 70 and above in the UK benefit from this research, and excludes the benefits for younger age groups, and the potential impact such a trial could have on global health, particularly in a pre-hospital context. Second, we used TBI incidence estimate from 2002/2003, which is likely an underestimate [34]. Lastly, it is based on the treatment effect risk ratio of 0.7 (0.45–1.08) derived from the CRASH-3 trial, which likely underestimates the uncertainty around the treatment effect in this population. When considering more modest mortality treatment effects (risk ratio of 0.8 to 0.95), the EVPI is much higher (Fig. 4). The cost-effectiveness of treatment becomes more uncertain as the proportion of simulations which have a treatment effect risk ratio favouring no tranexamic acid (risk ratio above 1) increases, therefore meaning the consequences associated with making the wrong decision become more likely, and thus, there is a higher value placed upon research to ensure the correct treatment decision is made.

### Limitations

Our base case results are based on the assumption that tranexamic acid is likely to reduce all-cause mortality. Although mild TBI patients with intracranial bleeding appear to benefit from tranexamic acid, there is no empirical evidence available to inform the average treatment effect for all mild TBI patients, or the level of uncertainty around it. Furthermore, the 0.5-g dose of tranexamic acid administered by an intramuscular injection planned for the CRASH-4 trial is lower than that used in the CRASH-3 trial, meaning there is additional uncertainty in the treatment effect and potential for adverse events [15]. However, such additional uncertainty would only increase the value of additional research. Eliciting expert opinion on the expected treatment effect could have provided an alternative estimate of the treatment effect.

There is also uncertainty around whether any additional dose of tranexamic acid would be administered as part of the standard of care, for those individuals with evidence of intracranial bleeding and receiving a scan within 3 h of their injury. Despite the CRASH-3 trial results demonstrating its effectiveness, NICE guidelines have not yet been updated and therefore a recommendation for its use in this indication has not been made.

Another limitation is that our study lacks an expected value of sample information (EVSI), to ensure the CRASH-4 trial design is justified from an economic perspective, and inform the optimal trial sample size [17]. However, given there is no empirical data on the treatment effect in this indication as yet, an EVSI analysis to inform the trial sample size might be most appropriate when an empirical estimate of the treatment effect, and the uncertainty around it is available. Ideally this would use the results of the pilot study, if these are unblinded and become available. This is particularly relevant given there are several limitations whereby the value of further research is likely underestimated in our analysis.

Our analysis also only included the cost of adverse events and did not consider any utility decrement, although this would be captured in the patient outcomes following TBI. If tranexamic acid is associated with an increase in adverse events in this indication, then this would reduce cost-effectiveness, but more importantly would raise questions about the risk to benefit ratio of treatment at an individual level.

Another limitation is that the model used data from multiple studies, with differing inclusion criteria and from various countries. As such, some of the data used may not be representative of older adults sustaining mild TBI in the UK.

Finally, the model did not capture the link between head injury and long-term health impacts, such as dementia. This is unlikely to bias our results unless tranexamic acid has an impact on the risk of dementia.

### Future research

Should the CRASH-4 trial proceed beyond the pilot phase, an updated economic analysis using the results of the trial would provide more certainty around whether tranexamic acid in a pre-hospital setting is cost-effective for mild TBI patients.

Research into how intramuscular tranexamic acid can be administered may also impact the cost-effectiveness. An auto-injector device is currently under development, which would reduce the time to treatment administration [36]. If an intramuscular injection of tranexamic acid is clinically effective, with evidence of a time to treatment effect, then an economic analysis could evaluate the cost-effectiveness of such as device compared to drawing up treatment at the scene.

Furthermore, if tranexamic acid is effective in mild TBI patients, then future studies should consider prognostic modelling to identify which patients are likely to benefit most from early treatment, and which are not, whilst also accounting for the cost-effectiveness. Older age, lower GCS scores (13-14) and anticoagulant use are all associated with higher mortality risk following mild TBI [4, 11, 14]. A prognostic model may help define a mortality risk “threshold” at which tranexamic is cost-effective, or identify subgroups of mild TBI patients who are more likely to benefit from treatment.

## Conclusion

If tranexamic acid reduces mortality in older people experiencing mild TBI, it is likely to be cost-effective. However, there remains a high degree of uncertainty around this, particularly due to the lack of evidence on the effectiveness of treatment in this specific population. From an economic perspective, the key parameters for collection in the CRASH-4 trial will be the tranexamic acid mortality treatment effect and the patient outcomes following TBI.

## Supplementary Information

**Additional file 1: Figure S1.** Markov Model structure. **Table S1.** Adverse event probabilities, tranexamic acid risk ratios and costs per event. **Table S2.** Deterministic cost-effectiveness results, by mean age. **Table S3.** Deterministic, univariate threshold analysis of potential treatment effects required for tranexamic acid to be cost-effective. For each treatment effect considered, the other potential treatment effects were not included (i.e. the parameters were the same for tranexamic acid and no tranexamic acid). **Figure S2.** A threshold analysis of the risk ratio treatment effect required for tranexamic avid to be cost-effectiveness across a range of mortality risks. Table S4: Expected value of perfect information across different time horizons. **Figure S3.** Expected value of partial perfect information (EVPPI) for groups of parameters. **Figure S4.** Proportion of cohort alive in first year following mild TBI (top) and over model time horizon (bottom) for no tranexamic acid and tranexamic acid treatment groups.

## Data Availability

Data from the CRASH-3 trial, which was used to inform some of the parameters in this analysis, is available from The Free Bank of Injury and Emergency Research Data (freeBIRD) website, a data sharing portal. The economic model code is available online (https://github.com/jackrdwilliams/CRASH-Mild-Model), and all data are reported in the manuscript.
