# Supplementary material for: A cost-effectiveness and value of information analysis to inform future research of tranexamic acid for older adults experiencing mild traumatic brain injury
Source: Trials. 2022 May 3;23:370. doi: 10.1186/s13063-022-06244-6 (PMC9066715; doi:10.1186/s13063-022-06244-6)
Supplement: Supplementary file 1 — Additional file 1: Figure S1. Markov Model structure. Table S1. Adverse event probabilities, tranexamic acid risk ratios and costs per event. Table S2. Deterministic cost-effectiveness results, by mean age. Table S3. Deterministic, univariate threshold analysis of potential treatment effects required for tranexamic acid to be cost-effective. For each treatment effect considered, the other potential treatment effects were not included (i.e. the parameters were the same for tranexamic acid and no tranexamic acid). Figure S2. A threshold analysis of the risk ratio treatment effect required for tranexamic avid to be cost-effectiveness across a range of mortality risks. Table S4: Expected value of perfect information across different time horizons. Figure S3. Expected value of partial perfect information (EVPPI) for groups of parameters. Figure S4. Proportion of cohort alive in first year following mild TBI (top) and over model time horizon (bottom) for no tranexamic acid and tranexamic acid treatment groups. [file 13063_2022_6244_MOESM1_ESM.docx]

**A cost-effectiveness and value of information analysis to inform future research of tranexamic acid for older adults experiencing mild traumatic brain injury.**

**Supplementary information**

Appendix Figure 1: Markov Model structure


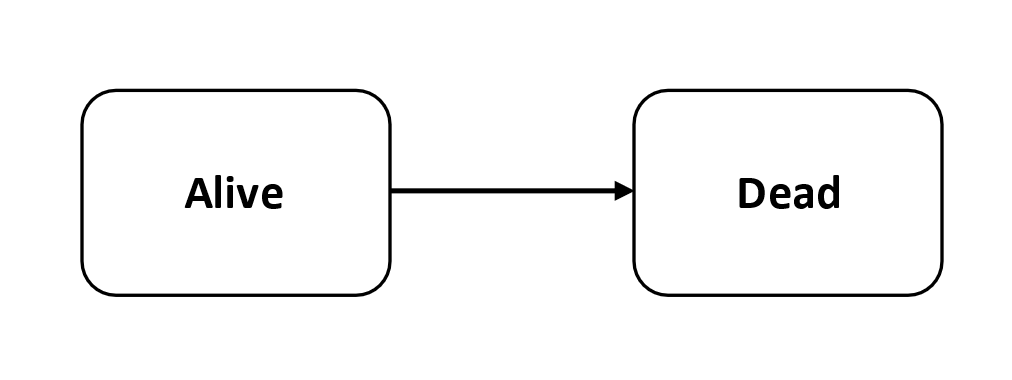


Appendix Table 1: Adverse event probabilities, tranexamic acid risk ratios and costs per event

| **Event** | **Probability (No tranexamic acid)** | **Probability Distribution** | **Tranexamic acid - Risk ratio** | **Risk ratio Distribution** | **Cost** |
| --- | --- | --- | --- | --- | --- |
| Pulmonary embolism | 0.40% | Beta(α=5, β=1247) | 0.978 | Lognormal (95% CI: 0.509-1.877) | £494.06 |
| Deep vein thrombosis | 0.08% | Beta(α=1, β=1251) | 1.222 | Lognormal (95% CI: 0.573-2.608) | £92.41 |
| Stroke | 0.16% | Beta(α=2, β=1250) | 1.233 | Lognormal (95% CI: 0.714-2.128) | £731.99 |
| Myocardial infarction | 0.32% | Beta(α=4, β=1248) | 0.733 | Lognormal (95% CI: 0.309-1.739) | £1,296.30 |
| Renal failure | 0.56% | Beta(α=7, β=1246) | 1.275 | Lognormal (95% CI: 0.902-1.802) | £464.40 |
| Sepsis | 2.40% | Beta(α=30, β=1222) | 1.037 | Lognormal (95% CI: 0.885-1.215) | £386.67 |
| Seizure | 1.20% | Beta(α=15, β=1237) | 1.211 | Lognormal (95% CI: 0.939-1.561) | £556.07 |
| Gastrointestinal bleeding | 0.48% | Beta(α=6, β=1246) | 0.711 | Lognormal (95% CI: 0.374-1.352) | £363.26 |

Source: The probability of adverse events in the control arm (placebo) was derived from mild TBI patients in the CRASH-3 study. The risk ratio of adverse events was derived from all patients in the CRASH-3 trial. The cost of each adverse event were derived from NHS reference costs.^1,2^

Appendix Table 2: Deterministic cost-effectiveness results, by mean age

| **Analysis** | **Treatment** | **Costs** | **QALYs** | **ICER (QALY)** |
| --- | --- | --- | --- | --- |
| 60 years old | No tranexamic acid | £29,904 | 9.1108 |  |
|  | Tranexamic acid | £30,090 | 9.1696 | £3,168 |
| 70 years old | No tranexamic acid | £23,035 | 5.9519 |  |
|  | Tranexamic acid | £23,177 | 5.9903 | £3,695 |
| 90 years old | No tranexamic acid | £10,424 | 1.1614 |  |
|  | Tranexamic acid | £10,485 | 1.1689 | £8,076 |

Appendix Table 3: Deterministic, univariate threshold analysis of potential treatment effects required for tranexamic acid to be cost-effective. For each treatment effect considered, the other potential treatment effects were not included (i.e. the parameters were the same for tranexamic acid and no tranexamic acid)

|  | **Treatment effect for tranexamic acid to be cost-effective at £20,000/QALY willingness to pay threshold** | | | |
| --- | --- | --- | --- | --- |
| **Age** | **Tranexamic acid mortality risk ratio** | **Utility increment for tranexamic acid versus no tranexamic acid (applied for 1 month only)^*^** | **Mean reduction in length of stay (days)^*^** | **Neurosurgery risk ratio^*^** |
| 60 | 0.998 | 0.00417 | 0.021 | 0.974 |
| 70 | 0.997 |  |  |  |
| 80 (base case) | 0.993 |  |  |  |
| 90 | 0.979 |  |  |  |

^*^The starting age of the cohort only influenced the tranexamic acid mortality risk ratio treatment effect required.

*Appendix Figure 2: A threshold analysis of the risk ratio treatment effect required for tranexamic avid to be cost-effectiveness across a range of mortality risks*


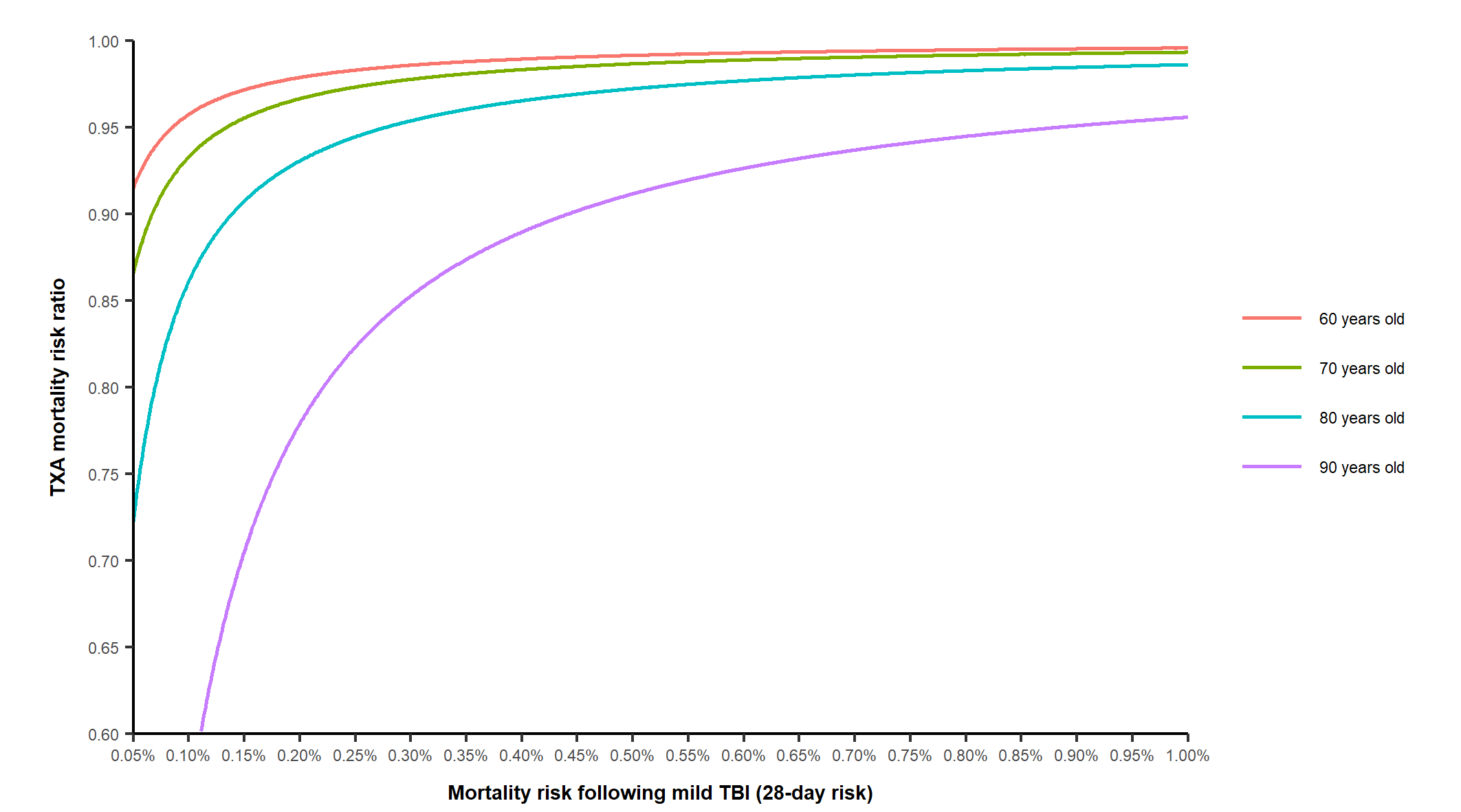


Appendix Table 4: Expected value of perfect information across different time horizons

| **Time Horizon (years)** | **Expected value of perfect information** |
| --- | --- |
| 5 | £6,274,091 |
| 10 | £12,031,074 |
| 20 (base case) | £22,357,773 |
| 30 | £30,551,121 |

Appendix Figure 3: Expected value of partial perfect information (EVPPI) for groups of parameters


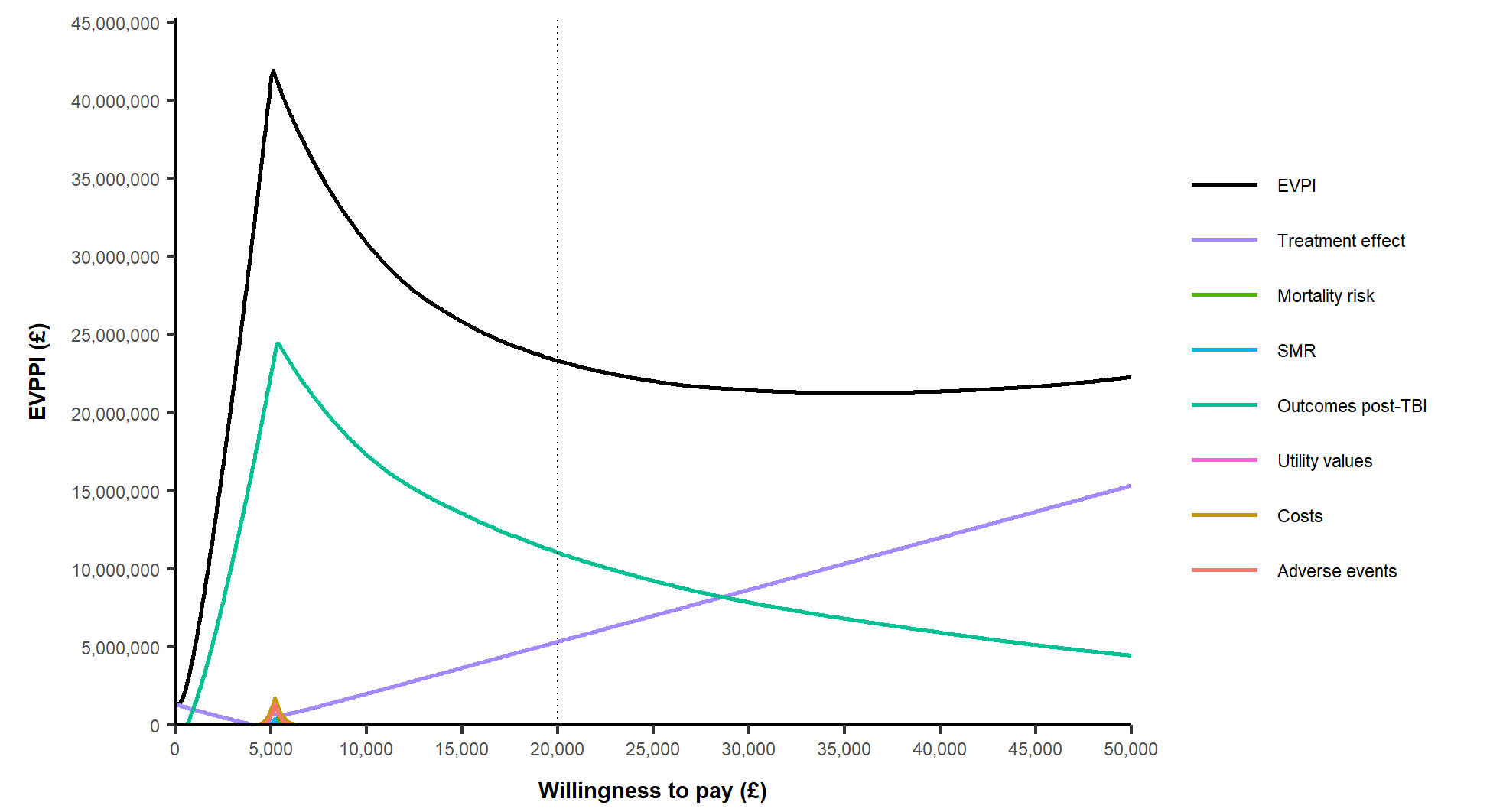


Appendix Figure 4: Proportion of cohort alive in first year following mild TBI (top) and over model time horizon (bottom) for no tranexamic acid and tranexamic acid treatment groups


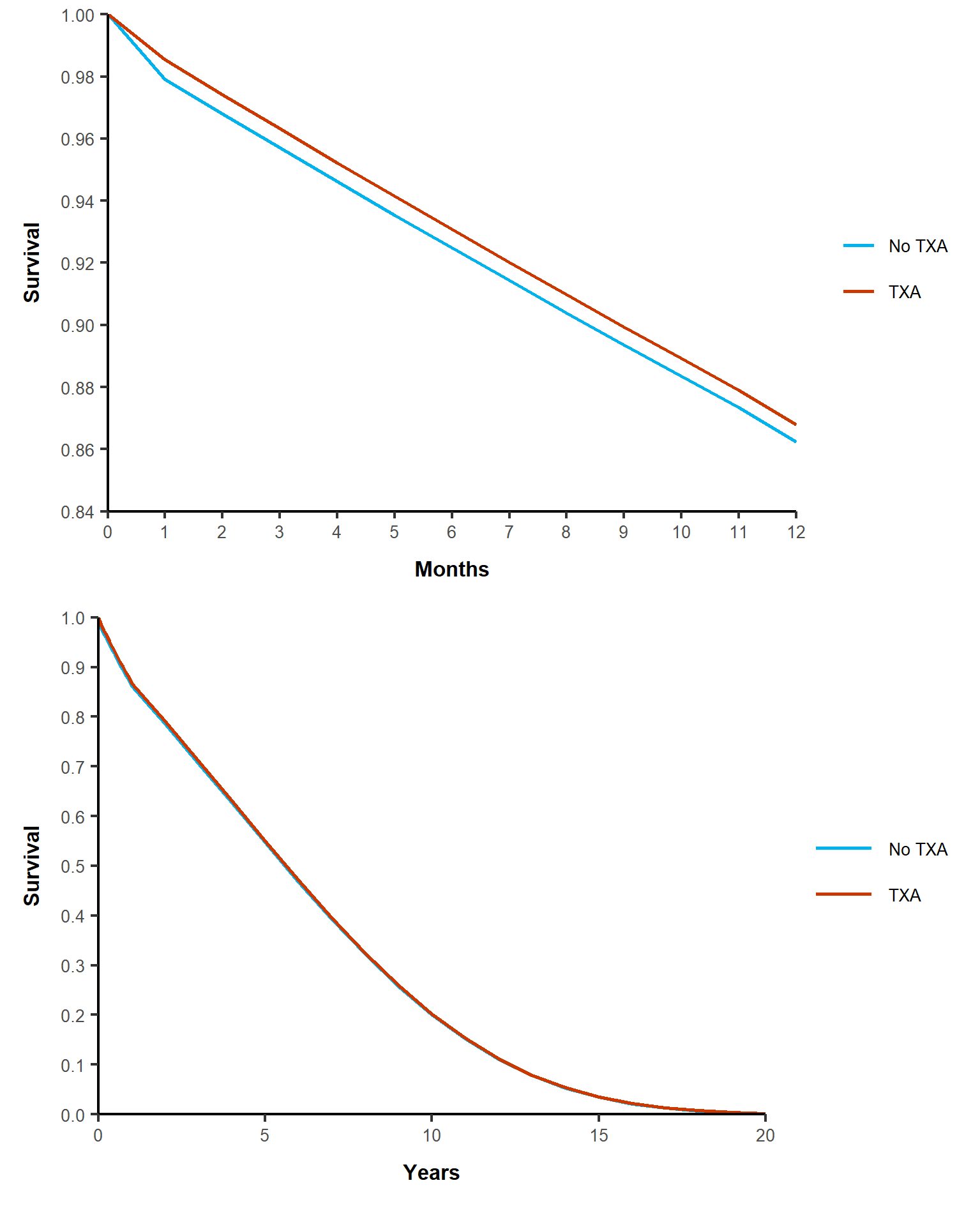


# References

1. The CRASH-3 trial collaborators. Effects of tranexamic acid on death, disability, vascular occlusive events and other morbidities in patients with acute traumatic brain injury (CRASH-3): a randomised, placebo-controlled trial. Lancet. 2019;394:1713-23.

2. NHS. National schedule of reference costs 2017-18. 2018.
